# Optogenetic Hyperpolarization of Cardiomyocytes Terminates Ventricular Arrhythmia

**DOI:** 10.3389/fphys.2019.00498

**Published:** 2019-04-24

**Authors:** Maximilian Funken, Daniela Malan, Philipp Sasse, Tobias Bruegmann

**Affiliations:** ^1^Institute of Physiology I, Medical Faculty, University of Bonn, Bonn, Germany; ^2^Department of Internal Medicine II, University Hospital Bonn, University of Bonn, Bonn, Germany; ^3^Research Training Group 1873, University of Bonn, Bonn, Germany; ^4^Institute of Cardiovascular Physiology, University Medical Center, Georg-August-University Göttingen, Göttingen, Germany; ^5^DZHK (German Research Centre for Cardiovascular Research), Partner Site Göttingen, Göttingen, Germany

**Keywords:** optogenetics, archaerhodopsin, ventricular arrhythmia, defibrillation, hyperpolarization, electrophysiology, ventricular tachycardia, ventricular fibrillation

## Abstract

Cardiac defibrillation to terminate lethal ventricular arrhythmia (VA) is currently performed by applying high energy electrical shocks. In cardiac tissue, electrical shocks induce simultaneously de- and hyperpolarized areas and only depolarized areas are considered to be responsible for VA termination. Because electrical shocks do not allow proper control over spatial extent and level of membrane potential changes, the effects of hyperpolarization have not been explored in the intact heart. In contrast, optogenetic methods allow cell type-selective induction of de- and hyperpolarization with unprecedented temporal and spatial control. To investigate effects of cardiomyocyte hyperpolarization on VA termination, we generated a mouse line with cardiomyocyte-specific expression of the light-driven proton pump ArchT. Isolated cardiomyocytes showed light-induced outward currents and hyperpolarization. Free-running VA were evoked by electrical stimulation of explanted hearts perfused with low K^+^ and the K_ATP_ channel opener Pinacidil. Optogenetic hyperpolarization was induced by epicardial illumination, which terminated VA with an average efficacy of ∼55%. This value was significantly higher compared to control hearts without illumination or ArchT expression (*p* = 0.0007). Intracellular recordings with sharp electrodes within the intact heart revealed hyperpolarization and faster action potential upstroke upon illumination, which should fasten conduction. However, conduction speed was lower during illumination suggesting enhanced electrical sink by hyperpolarization underlying VA termination. Thus, selective hyperpolarization in cardiomyocytes is able to terminate VA with a completely new mechanism of increased electrical sink. These novel insights could improve our mechanistic understanding and treatment strategies of VA termination.

## Introduction

Electrical shocks are the only acute life-saving treatment option for patients with ventricular tachycardia or ventricular fibrillation (ventricular arrhythmia, VA) and subsequent loss of cardiac output (Moss et al., 1996). Electrical shocks consist of one mono- or biphasic electrical field stimulation with high energy applied from external or implanted cardiac defibrillators in order to terminate the underlying high frequency activation and restore sinus rhythm. Due to the anisotropic architecture of the heart with different electrical properties of the intracellular versus the extracellular compartment (Clerc, 1976; Corbin and Scher, 1977; Roberts et al., 1979), the electrical stimulation induces simultaneously de- and hyperpolarized areas (Roth, 1994). These so-called virtual electrodes (Wikswo et al., 1995) consist of a virtual cathode with depolarization in a dog bone shape (Akar et al., 2001) and a perpendicular virtual anode with hyperpolarization parallel to the fiber orientation (Knisley, 1995; Neunlist and Tung, 1995; Sambelashvili et al., 2003) which can be even larger than the virtual cathode (Nikolski et al., 2004). For decades successful defibrillation has only been attributed to the depolarized tissue while the hyperpolarization is considered to generate new wavefronts by a anode-break mechanism (Cranefield et al., 1957) or create a phase singularity which can trigger a new excitation and generate a new arrhythmic wavefront (Efimov et al., 1998; Roth, 1998; Charteris and Roth, 2011). However, the specific effects of hyperpolarization in the intact heart could not be addressed experimentally so far because it is impossible to predict or control the extent of the virtual electrodes induced by electrical field stimulation.

Optogenetic stimulation enables selective hyperpolarization with light within an intact organ in a cell type of interest expressing light-inducible pumps (Wiegert et al., 2017). To analyze the effects of selective hyperpolarization in the intact heart, we expressed the light-driven proton pump archaerhodopsin from the Halorubrum sodomense strain TP009 (ArchT) (Han et al., 2011) selectively in cardiomyocytes in a transgenic animal model. ArchT transports H^+^ outside of the cell upon illumination with green light with a peak wavelength of ∼550 nm leading to hyperpolarization of the cell-membrane (Mattis et al., 2012). The cellular buffer capacity prevents the cells from alkalosis if ArchT is activated only for a few seconds (Chow et al., 2010; Mahn et al., 2016). Importantly being an unidirectional outward pump, ArchT induces hyperpolarization without a reversal potential, which is an advantage over optogenetic Cl^−^ or K^+^ conducting ion channels, which are ineffective or even depolarizing at membrane potentials close or below their reversal potential, respectively (Govorunova et al., 2015; Alberio et al., 2018; Bernal Sierra et al., 2018). Specifically, the recently identified Cl^−^ selective channelrhodopsin variants are not suited because the high intracellular Cl^−^ concentration results in light-induced depolarization in cardiomyocytes as elegantly described by Kopton et al. (2018) in this focused issue. Importantly, optogenetic hyperpolarization of cardiomyocytes has been used before *in vitro* by co-culture with ArchT-expressing fibroblasts (Nussinovitch et al., 2014) and in ArchT-expressing human induced pluripotent stem cell-derived cardiomyocytes (Quach et al., 2018).

Thus in this project, we aimed to detect the effects of sole hyperpolarization in the intact heart and to decipher a potential role in termination of VA by using ArchT expression in cardiomyocytes.

## Materials and Methods

An expanded Methods section is available in Supplementary Material online. All animal experiments were performed in accordance to the European Guideline for animal experiments 2010/63/EU. Ethical approval for animal experiments was not required because experiments were exclusively performed *ex vivo* on isolated hearts and transgenic animals did not show any pathological phenotype (as assessed by standardized score sheets for animal welfare). Mice were sacrificed by cervical dislocation.

### Mouse Model, Expression Analysis, and Patch Clamp

Transgenic mice were generated by crossbreeding αMHC-Cre (Agah et al., 1997) with Ai40D mice, which express ArchT in fusion to eGFP after Cre-mediated excision of a floxed stop cassette (Daigle et al., 2018). Light-induced outward currents and their impact on membrane potential and action potentials (AP) were determined using whole cell patch-clamp recordings (Bruegmann et al., 2010; Vogt et al., 2015). ArchT was activated through the objective with green light (520 nm).

### Optogenetic Defibrillation

Free-running sustained VA were induced by epicardial burst (50 Hz) or S1-S2 electrical stimulation (2 ms, 1–10 mA) of explanted hearts perfused with Tyrode solution containing 2 mM K^+^ and the K_ATP_-channel activator Pinazidil (100 μM) as reported previously (Bruegmann et al., 2016). The anterior ventricular wall was illuminated with a macroscope with green light (525 nm). Efficacy of optogenetic VA termination were analyzed with a 11 s long protocol with 4 light pulses (see Supplementary Figure 1B) and compared to spontaneous VA termination in control groups within the exact same time window.

### Sharp Electrode Measurements in Intact Hearts

Cardiomyocytes’ AP were recorded in explanted hearts perfused with Tyrode solution containing 2 mM K^+^, 100 μM Pinacidil, as well as Blebbistatin (10 μM) and 2,3-Butanedione monoxime (7.5 mM) to inhibit contractions. Microelectrodes (filled with 3 M KCl, 60 – 120 MΩ,) were penetrated with a fast piezo actuator (5–10 μm steps) until stable resting membrane potential < −60 mV was obtained. Hearts were electrically paced using silver chloride electrodes placed < 3 mm from the recording site. For each recording site, AP upstroke velocity and conduction time were normalized to maximal values.

### Statistics

Data are shown as mean ± s.e.m. Statistical analyses were performed using GraphPad Prism with the one-way ANOVA Kruskal–Wallis and Dunn’s multiple comparison post-test for VA termination rates, two-sided paired students *t*-test for patch clamp experiments and a 2-way-ANOVA repeated measurements test for sharp electrode experiments. A *p*-value <0.05 was considered statistically significant.

## Results

**FIGURE 1:**
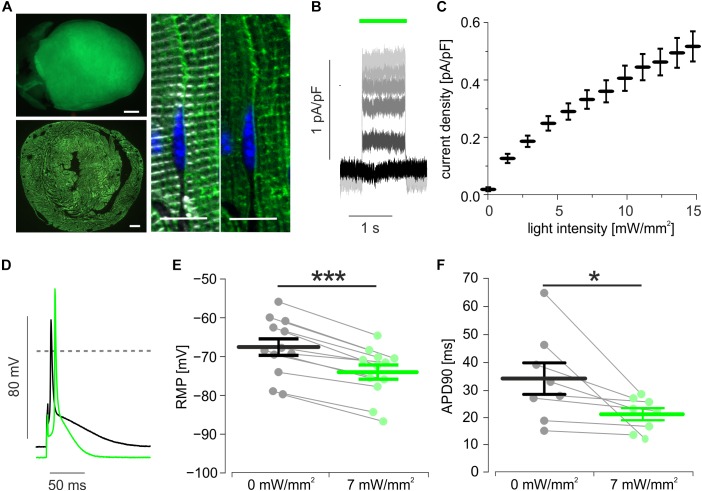
Expression and function of ArchT in isolated cardiomyocytes. **(A)** eGFP fluorescence (green) of an ArchT-eGFP expressing mouse heart (**A**, top, bar = 1 mm), of a ventricular section (**A**, low, bar = 500 μm), and in α-actinin (white) positive cardiomyocytes (**A**, right, bar = 10 μm, nuclear staining in blue). **(B)** Representative traces of outward currents induced by illumination (green bar) with increasing light-intensities (from black to light gray: 0, 3, 6, 8, 10, 14 mW/mm^2^). **(C)** Statistical analysis of light intensity to current density relationship (*n* = 14 cells). **(D)** Representative AP before (black) and during illumination (green, 7 mW/mm^2^). **(E,F)** Statistical analysis of light-induced changes in resting membrane potential (**E**, *p* < 0.0001, *n* = 12 cells) and AP duration (**F**, *p* = 0.039, *n* = 8 cells) using two-sided paired students *t*-test. ^∗^*p* < 0.05; ^∗∗∗^*p* < 0.001.

Transgenic mice expressing ArchT-eGFP showed bright eGFP fluorescence signals on the epicardial surface which were homogenously distributed throughout the whole myocardial wall and were restricted to the plasma membrane and t-tubulus invaginations of cardiomyocytes (Figure 1A). Single cell dissociation revealed 97.4 ± 0.2% (*n* = 4) eGFP positive cardiomyocytes, which showed green (520 nm) light-evoked outward currents (Figure 1B) with current density depending almost linearly on the light intensity (Figure 1C). During current clamp recording of stimulated AP, illumination (7 mW/mm^2^) led to hyperpolarization by 6.5 ± 0.7 mV (Figure 1D,E) and AP duration (APD) shortening by 24.3 ± 5.8% (Figure 1D,F).

**FIGURE 2:**
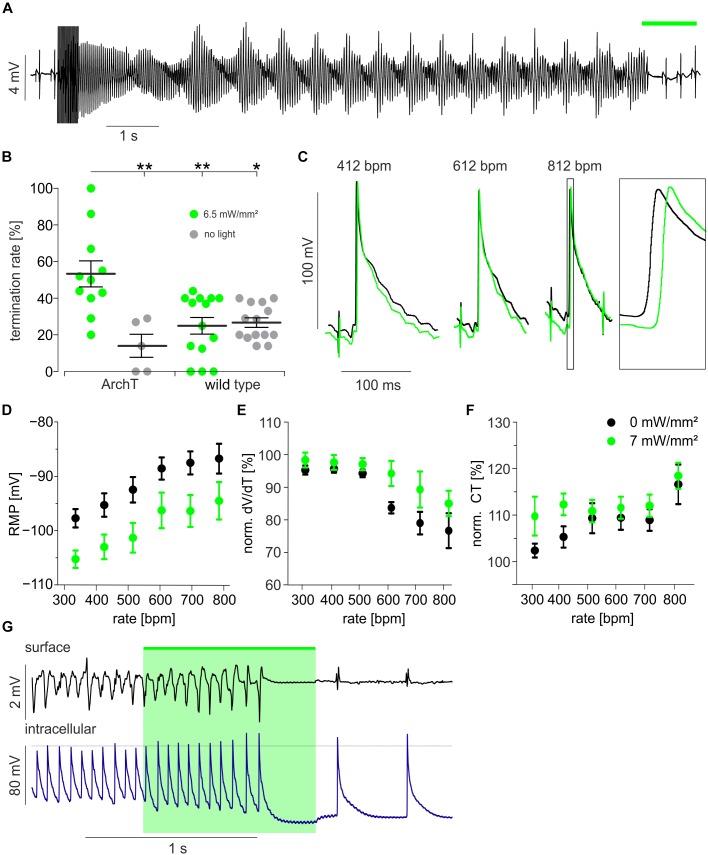
Optogenetic defibrillation by light-induced hyperpolarization. **(A)** Representative example of a Torsade-de-pointe like ventricular arrhythmia induced by electrical burst stimulation and terminated by illumination (**green bar**, 3.4 mW/mm^2^) of the ventricular surface (24 mm^2^). **(B)** Statistical analysis of termination rates in ArchT expressing (*n* = 11 and 5) and wild type mice (*n* = 14) with (green) and without (gray) illumination (see section “Materials and Methods” and Supplementary Figure 1B) using the one-way ANOVA Kruskal–Wallis test with Dunn’s multiple comparison post-test (^∗^*p* < 0.05; ^∗∗^*p* < 0.01). **(C–F)** Sharp electrode measurements within the illuminated region of the intact heart during electrical pacing from a distant site (see Supplementary Figure 1C): Original traces of AP recorded while pacing with 412, 612, and 812 bpm before (black) and during illumination (green, 6.5 mW/mm^2^, 24 mm^2^) **(C)**. Analysis of resting membrane potential (RMP) (**D**, *n* = 4, *p* < 0.0001), max. AP upstroke velocity (dV/dt) (**E**, *n* = 4 – 10, *p* < 0.0001) and conduction time (CT) from the electrical stimulus to the AP generation (**F**, *n* = 4 – 10, *p* = 0.01) before (black) and during illumination (green) (two-way ANOVA repeated measurements test, *p*-values are indicating the influence of light). **(G)** Cardiac surface electrogram and simultaneous intracellular membrane potential recording during a ventricular arrhythmia episode with termination by light (green, 6.5 mW/mm^2^, 24 mm^2^).^∗^*p* < 0.05; ^∗∗^*p* < 0.01.

To assess the effects of hyperpolarization on arrhythmia termination, hearts were explanted and perfused retrogradely in the Langendorff configuration (Supplementary Figure 1A). To obtain free-running sustained VA in the small mouse heart, extracellular K^+^ concentration was reduced to 2 mM and the K_ATP_ channel opener Pinacidil was applied in order to decrease the cardiac wavelength by conduction slowing and AP shortening (Bruegmann et al., 2016, 2018). Electrical burst or S1 S2 stimulation protocols evoked VA consisting of ventricular tachycardia including Torsade-de-Pointe like arrhythmia (Figure 2A) and ventricular fibrillation. Illumination (525 nm, 1 s) of the anterior ventricular surface (24 mm^2^) terminated VA inconsistently in some but not during all attempts (Figure 2A). For exact determination of optogenetic VA termination efficacy, we performed a precisely timed four light pulse protocol (Supplementary Figure 1B). VA termination rates were determined in the same 11 s long time window in all hearts. This yielded an average VA termination success rate of 53 ± 2% for optogenetic hyperpolarization of ArchT expressing hearts. Importantly, VA termination rate was significantly lower in control non-illuminated and/or non-ArchT expressing hearts, which showed spontaneous VA termination rates <30% (Figure 2B).

To gain insights into the mechanism underlying hyperpolarization-induced VA termination, we recorded AP from cardiomyocytes (Supplementary Figure 1C) by impaling sharp microelectrodes within the illuminated (6.5 mW/mm^2^) area of intact hearts. Because of the high frequency of VA, we tried to mimic this situation by electrical pacing up to 812 bpm (Figure 2C). Pacing at higher heart rates increased the resting membrane potential and epicardial illumination reduced resting membrane potential similarly at all heart rates (Figure 2D). Interestingly, AP upstroke velocity was reduced at heart rates >600 bpm indicating relative refractoriness through reduced Na^+^ channel recovery from inactivation due to the depolarized resting membrane potential. This effect was partly reverted by light-induced hyperpolarization (Figure 2E). Light-induced restoration of Na^+^ channel availability at high heart rates *per se* should result in faster conduction of the electrical excitation wave through the ventricles. However, when analyzing the conduction time between the electrical pacing site and the AP initiation at the recording site, we observed the opposite effect: At all beating rates this parameter was larger during illumination indicating slower electrical conduction or a longer path length during illumination and the effect was most prominent at slow pacing rates (Figure 2F). This might be explained by an increased electrical sink upon light-induced hyperpolarization, resulting in slower AP conduction from cell to cell. Importantly during an episode of free-running VA, we observed light-induced hyperpolarization and shortening of APD which was accompanied with a decrease in VA complexity from polymorphic to almost monomorphic VA finally resulting in VA termination (Figure 2G). Thus, we conclude that the increased electrical sink is the most likely mechanism underlying hyperpolarization-induced VA termination in our model.

## Discussion

Using optogenetic stimulation, we were able to selectively induce hyperpolarization within the intact heart and to demonstrate that hyperpolarization *per se* can terminate VA. In general, VA are triggered by an ectopic premature ventricular excitation and maintained by a short cardiac wavelength (conduction velocity ^∗^ APD), which can be reduced by several additive mechanisms: The fast VA rate leads to (1) depolarization of the resting membrane potential (Figure 2D). In consequence, subthreshold depolarization can speed up propagation by bringing cells closer to the excitation threshold, which was shown elegantly by low dose optogenetic depolarization in a two dimensional monolayer of cardiomyocytes (Burton et al., 2015). However, further depolarization is eventually lowering Na^+^ channel availability resulting in (2) slow AP upstroke, and (3) reduced conduction velocity. Furthermore Ca^2+^ channels are inactivated leading to (4) shorter APD. Using sharp electrode experiments, we could confirm the effects (1), (2), and (3) at high pacing rates.

We found that optogenetic hyperpolarization reduces APD (Figure 1F), which would rather stabilize VA but could explain the reduction in complexity from polymorphic VA into VT (Figure 2G). In line with Na^+^ recovery by hyperpolarization, we observed faster AP upstroke velocities upon illumination, but this effect was not strong enough to decrease conduction time, e.g., by fasten conduction velocity. In contrast, the delay between the distant electrical pacing and AP generation was prolonged during illumination (Figure 2F) which can be explained by (A) delayed AP initiation at the pacing site, (B) lower conduction velocity between stimulation and the recording site, or (C) a different longer conduction pathway from the stimulation to the recording electrode. Importantly, all three effects would be indicators for an increased electrical sink pulling the resting membrane potential away from the excitation threshold (Burton et al., 2015). In consequence, hyperpolarized resting cardiomyocytes require more inward current from electrical pacing (A) or from the activated neighbor cardiomyocytes (B) to be depolarized above the AP threshold, which is important to maintain the arrhythmic wavefront. Unfortunately, using only one sharp electrode does not allow direct investigation of altered conduction pathways (C) by optogenetic hyperpolarization, however, solving this by combining non-transparent multi-electrode recording or spectrally overlapping voltage mapping with ArchT stimulation is technically challenging. We therefore conclude that the hyperpolarization-induced increase in electrical sink is the main mechanism terminating the VA episodes in our model, in which we have obtained stable VA using low K^+^ concentrations and APD shortening by opening K_ATP_ channels.

Compared to continuous optogenetic depolarization using Channelrhodopsin 2 with an efficacy >95% (Bruegmann et al., 2016), VA termination by ArchT-induced hyperpolarization had a much lower and variable efficacy of ∼55%. This can be explained by parallel pro-arrhythmic effects of ArchT activation such as shorter APD reducing the cardiac wavelength or Na^+^ channel restoration, which would increase the electrical source of the leading wavefront. Furthermore the low pumping rate of ArchT limits the amount of light induced outward current and hyperpolarization (Mahn et al., 2016) and we had to restrict light intensity to 6.5 mW/mm^2^ to avoid cellular damage.

Importantly, the low efficacy excludes optogenetic hyperpolarization from any translational outlook, at least using the ineffective proton pump ArchT. Further development of effective and fast optogenetic K^+^-selective channels could allow better insights into the efficacy of VA termination by increasing electrical sink. Selective hyperpolarization as well as combined optogenetic de- and hyperpolarization using spectrally separated optogenetic proteins will lead to a better understanding of VA maintenance and termination mechanisms and might improve current treatment strategies by electrical shocks and antiarrhythmic drugs.

## Conclusion

Cardiomyocyte-specific expression of the light-inducible proton pump ArchT allowed to investigate the effects of isolated hyperpolarization in the intact heart. Thereby we were able to demonstrate that hyperpolarization *per se* can terminate VA with an increased electrical sink being the most probable mechanism.

## Ethics Statement

Animal breeding and handling were performed in accordance to the European Guideline for animal experiments 2010/63/EU. Ethical approval for animal experiments was not required because experiments were exclusively performed *ex vivo* on isolated hearts and transgenic animals did not show any pathological phenotype (as assessed by standardized score sheets for animal welfare).

## Author Contributions

TB and PS designed the study. MF, DM, PS, and TB performed the experiments. MF, DM, and TB analyzed the data. MF, PS, and TB prepared the manuscript.

## Conflict of Interest Statement

The authors declare that the research was conducted in the absence of any commercial or financial relationships that could be construed as a potential conflict of interest.

## Abbreviations

aMHC: α-myosin-heavy-chain
AP: action potential
APD: action potential duration
ArchT: archaerhodopsin from Halorubrum strain TP009
VA: ventricular arrhythmia.
