# Supplementary material for: Optogenetic Hyperpolarization of Cardiomyocytes Terminates Ventricular Arrhythmia
Source: Front Physiol. 2019 Apr 24;10:498. doi: 10.3389/fphys.2019.00498 (PMC6491897; doi:10.3389/fphys.2019.00498)
Supplement: Supplementary file 1 [file Data_Sheet_1.PDF]

Supplementary Material

1 Online material and methods

Mouse model

Animal breeding and handling were performed in accordance to the European Guideline for animal experiments 2010/63/EU. Ethical approval for animal experiments was not required because experiments were exclusively performed *ex vivo* on isolated hearts and transgenic animals did not show any pathological phenotype (as assessed by standardized score sheets for animal welfare). Mice expressing ArchT and eGFP as fusion protein under control of the chicken-β-actin promotor after Cre-mediated removal of the floxed STOP cassette in cardiomyocytes were generated by crossbreeding αMHC-Cre mice (Agah, et al. 1997) with C57BL/6 background (crossbreeded with CD1 wildtype mice) and Ai40D mice (#021188, Jackson Lab, Bar Harbor, ME, USA (Daigle, et al. 2018) with C57BL/6J background (crossbreeded with CD1 wild type mice). Genotyping was performed by PCR using ATGACAGACAGATCCCTCCTATCTCC and CTCATCACTCGTTGCATCGAC primers for αMHC-Cre mice and GACGTCTCGTCGCTGATTGG and CGGGCCATTTACCGTCATTG primers for Ai40D mice. Phenotype was controlled by checking epicardial fluorescence signals of the heart prior to experiments (see Fig. 1A). For defibrillation experiments, 11 double transgenic mice (five male, six female, aged 4-10 months, mean 4.5 months) were used. 14 control animals (12 male, two female, aged 4-6.5 months, mean 6 months) were taken from the littermates of same breeding but without non-double transgenic genotype to ensure the same genetic background. Sharp electrode measurements were performed with hearts from four double transgenic female mice aged 4 months and patch clamp experiments of cardiomyocytes isolated from three double transgenic female mice aged 4 months.

Optogenetic defibrillation

Mice were sacrificed by cervical dislocation. After explantation, hearts were perfused in Langendorff configuration with normal Tyrode solution (in mM 140 NaCl, 5.4 KCl, 2 $MgCl_2$, 1.8 $CaCl_2$, 10 HEPES and 10 Glucose, pH 7.4 using NaOH). Two point electrograms were recorded using a silver chloride electrode in contact to the right atrium and a metal spoon stabilizing the apex of the heart. The signal was amplified with an animal bio-amplifier and a PowerLab recording system (ML 136, PowerLab 8/30, LabChart software ADInstruments, Sydney, Australia). As previously reported (Bruegmann, et al. 2016), we subsequently lowered the extracellular K^+ concentration to (KCl 2mM, compensatory NaCl 144 mM) and added the K_{ATP}–activator Pinacidil (100µM, Sigma-Aldrich, St. Louis, MO, USA) to reduce the cardiac wavelength and enable free-running VA in the small mouse heart. VA were induced by electrical burst stimulation (50 Hz, 20-50 biphasic 2ms pulses, 1–10 mA) via a bipolar silver chloride electrode (<3 mm distance) placed epicardially on right ventricular base of the heart. Alternatively, we used S1-S2 protocols with S2 exceeding local effective refractory period by 5-20 ms. Because self-termination of VA occurred mostly within the first three seconds, optogenetic defibrillation protocols were started at least three seconds after the electrical stimulation to exclude self-terminating VA from analysis (see Suppl. Fig 1B and legend).

Illumination was performed using a 525 nm LED (GCS-0525-60-A0510, Mightex, North York, Canada) coupled into the epifluorescence port of a macroscope (MVC10, Olympus, Shinjuku, Japan)

equipped with a 1x objective (MVPLAPO 1x, numerical aperture 0.25). Light was focused with 4x magnification on the anterior epicardium of the ventricles (Suppl. Fig. 1A). Illumination was controlled with the PowerLab system and light intensity was calibrated using a powermeter (PM100 with S130A sensor, Thorlabs, Newton, NJ, USA). To test efficacy of optogenetic hyperpolarization for VA termination, we performed a four-light-pulse protocol with 1 s long light pulses applied every 2 seconds and 4 s long pause after the last light pulse (Suppl. Fig. 1B). Only when VA terminated within this 11 s long time period, the termination attempt was considered successful. Every protocol (with and without illumination analyzed within the same time window) has been repeated at least five times in the same heart and the termination rate for each condition in every heart was averaged (indicated by one dot in Fig. 2B) and taken for statistical comparisons. If VA persisted after this protocol, we tried to restore sinus rhythm by using longer illuminations (6.5 mW/mm^2 for at least 3 s), antitachycardia pacing (50 Hz > 2s) and mechanical defibrillation by hitting the ventricles with tweezers (Suppl. Fig. 1B). If the combination of all three rescue procedures failed, we reperfused the hearts with standard Tyrode solution with normal K^+ concentration (5.4 mM) and without Pinacidil.

Patch clamp experiments

Cardiomyocytes were isolated from double transgenic mice as described previously (Bruegmann, et al. 2010, Vogt, et al. 2015, Fehrentz et al. 2018,). Briefly, hearts were perfused in Langendorff configuration with Tyrode solution (in mM: 135 NaCl, 4 KCl, 1 $MgCl_2$, 2.5 HEPES, 5 glucose, 25 butanedione monoxime; pH 7.4) for 5 min at 37 °C and subsequently with the same Tyrode solution containing also 50 µM $CaCl_2$, 1.0 mg/ml collagenase B (Hofmann-la Roche, Basel, Switzerland) and 0.1 mg/ml trypsin (Sigma-Aldrich) for 13–14 min. Ventricles were cut in small pieces and mechanically dissected; cells were filtered through a nylon mesh (100µm) and the pellet was resuspended in Tyrode solution with 50 µM $CaCl_2$ and 5% FCS. Afterwards, Ca^{2+} was increased in four steps to 1.8 mM over 40 min.

For characterization of ArchT function in single adult cardiomyocytes, we performed electrophysiological recordings by patch clamp technique. Single cells were plated at low density on laminin-coated (0.1%) coverslips in external solution containing (in mM) 140 NaCl, 5.4 KCl, 1.8 $CaCl_2$, 1 $MgCl_2$, 10 HEPES, 10 glucose, pH 7.4 (NaOH). Patch-clamp experiments were performed using an EPC10 amplifier and the Patchmaster software (Heka, Ludwigshafen, Germany) in the whole cell configuration. Pipette solution contained (in mM): 50 KCl, 80 K-Asparatate, 1 $MgCl_2$, 3 MgATP, 10 EGTA, 10 HEPES, pH 7.4 (KOH). Illumination was performed with a 500-600 nm broadband LED within the LED Hub (Omicron, Rodgau, Germany) and a 520/35 nm filter (AHF Analysetechnik, Tübingen, Germany) controlled by the EPC10 amplifier and coupled to the epifluorescence port of an inverted microscope (Axiovert 200, 20x Fluar objective, NA: 0.75, Zeiss, Oberkochen, Germany). Light-induced currents were recorded in voltage clamp mode at a sampling rate of 20 kHz and 1 kHz filter. Cardiomyocytes were held at a holding potential of −60 mV for 1000 ms, followed by a 1000 ms step with light application at different intensities from 0 mW/mm^2 to 14 mW/mm^2 (~ 1 mW/mm^2 steps every 2 s). The steady-state currents were analyzed by averaging the currents between 500 to 970 ms of the light pulse using Fitmaster Software (Heka) and subtracting the currents during the 840 ms prior to the illumination. Resting membrane potential and AP were recorded in the current clamp mode at a sampling rate of 5 kHz and a 1 kHz filter. AP were elicited at 1 Hz by a 2 ms current injection pulse of 700-1000 pA through the patch pipette. AP were stimulated five times per sweep with and without illumination throughout the whole sweep. Resting membrane potential and AP duration at 90% of repolarization (APD90) were analyzed with the cardiac AP analysis module of the Labchart software.

Sharp electrode recordings

Cardiomyocytes' syncytial membrane potential was measured within explanted hearts perfused with the same Tyrode as for the defibrillation experiments containing low potassium (2 mM) and Pinacidil (100 µM) with additional Blebbistatin (10 µM, Enzo Life Science, Lörrach, Germany) and 2,3-Butanedione monoxime (7.5 mM, Sigma-Aldrich) to inhibit contractions. Sharp microelectrodes (1B100F-4, World Precision Instruments, Sarasota, FL, USA) were pulled with micropipette-puller (Model P1000, Sutter Instruments, Novato, CA, USA) and filled with 3M KCl (60-120 MΩ resistance). The left ventricular free wall (Suppl. Fig. 1C) was impaled with a piezo crystal equipped manipulator (DC3001 micromanipulator with piezo-translator SYS-MPM10, World Precision Instruments, Sarasota, Florida, USA) using 5-10 µm steps at ~100 mm/s piezo speed. Intracellular recordings were amplified with a bridge amplifier (BA-03X, NPI electronics, Tamm, Germany) and recorded using the Powerlab system simultaneously with the surface electrograms from the heart. Stable intracellular recordings from cardiomyocytes were identified by a resting membrane potential lower than −60 mV and AP peaks above 0 mV. Hearts were paced continuously after impalement using the silver chloride electrodes (2 ms, biphasic, 1-10 mA) at frequencies between 312 and 812 bpm. We performed 5 s of illumination with 5 s interval in-between with the same illumination setup as described above. Resting membrane potential and AP upstroke velocity were analyzed with LabChart using the peak detection module for cardiac AP. Resting membrane potential was defined as lowest value of the membrane potential between AP. To quantify conduction, we measured the conduction delay from the pacing electrodes (identified by the stimulation artefact) to AP initiation at the site of the impaled sharp electrode (determined by the time point of fastest AP upstroke velocity). Because of high variation, we normalized values to the lowest conduction delay and fastest AP upstroke velocity of each cell.

Expression analysis

Pictures of dissociated cardiomyocytes were taken with a fluorescence microscope (Axiovert 200M, Zeiss) with a 10x objective (10x, NA 0.3, Zeiss) and the F51-024 FITC/TRITC filterset (Zeiss). Percentage of ArchT expressing cardiomyocytes were determined by manually counting eGFP positive and total cell numbers in ten randomly chosen fields of view per heart using the ImageJ software.

Epicardial fluorescence pictures of explanted hearts were acquired using a macroscope (AxioZoom V16, Zeiss) equipped with a PlanApoZ ×1.0 objective lens and a calibrated light source (HXP 200 C, Zeiss), an GFP filterset (F46-002, AHF Analysetechnik) and the AxioCam MRm camera (Zeiss). Pictures were recorded and processed using the Zen 2012 software (Zeiss) using Z-stacks and the extended focus module.

For immunohistochemistry, explanted hearts were perfused in Langendorff configuration with 13 ml of 4% formaldehyde solution and stored in the same solution for 24 h at 4° C. Afterwards the hearts where washed multiple times in PBS, dehydrated in 20% sucrose for >24 h and subsequently frozen in Tissuetec matrix gel (Sakura Europe, Staufen, Germany) at − 80° C. Hearts were sectioned with a Cryostat 3050 S (Leica, Wetzlar, Germany) into 10 µm thick sections. Staining was performed as reported earlier (Bruegmann, et al. 2010). Briefly, sections were permeabilized with 0.2% Triton X (Sigma-Aldrich) for 20 minutes, blocked with 5% donkey serum (Jackson ImmunoResearch, West Grove, PA, USA) for 2 h at room temperature and stained overnight at 4° C with primary antibody for α-actinin (1:400, Sigma-Aldrich). Cy5 conjugated secondary antibodies (1:400, Jackson ImmunoResearch) diluted in 0.1% Hoechst 33342 (Sigma-Aldrich) were applied for 1 h at room temperature. Overview-pictures of the stained slices were taken using the same AxioZoom V16

macroscope. Magnification pictures were taken with an Axiovert 200M microscope equipped with the Apotome section module (Zeiss) at 63x magnification (Plan-Apochromat 63x Oil objective, NA 1.4, Zeiss), eGFP and Cy5 filter set (AHF Analysetechnik). Pictures were processed using the Axiovision software (Zeiss).

Statistics

Data are shown as mean ± s.e.m.. Statistical analyses were performed using GraphPad Prism (GraphPad Software, San Diego, CA, USA). Termination rates with and without illumination in ArchT expressing versus wild type hearts (Fig. 2B) were compared using the one-way ANOVA Kruskal-Wallis with Dunn's multiple comparison post-test because of the non-normal distribution and unequal variances of average termination rates. Results from sharp electrode experiments (Fig. 2D-F) were tested using a repeated measurements 2-way-ANOVA for the effect of light. Patch clamp experiments (Fig. 1E,F) were analyzed with two-sided, paired students t-test. A p-value < 0.05 was considered statistically significant.

2 Supplementary Figures and Tables

2.1 Supplementary Figure

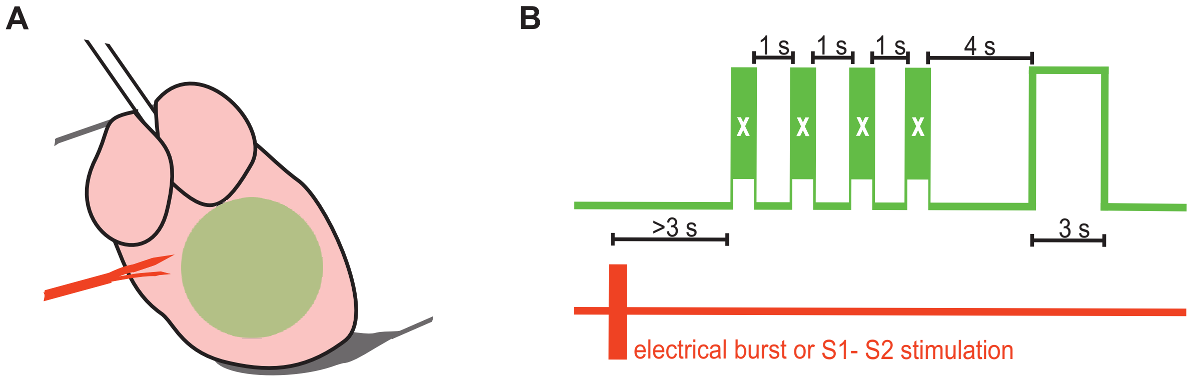

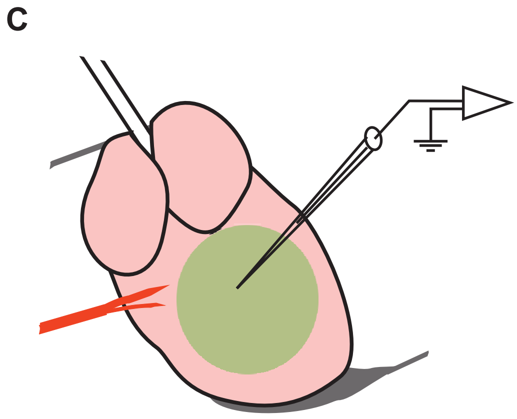

Supplementary Figure 1: Schematic overview of optogenetic defibrillation experiments by hyper-polarization and sharp electrode measurements. (**A**) Hearts were explanted and perfused in the Langendorff configuration. Electrical stimulation was performed using two silver electrodes (**red**) placed on the right ventricular base of the heart. Illumination was focused on the anterior epicardial surface of both ventricles (**green circle**, 24 mm^2). (**B**) Illumination protocol to test the efficacy of optogenetic defibrillation. We applied electrical (**red**, 2 ms, biphasic 1 to 10 mA) burst (20-50 times, 50 Hz) or S1 S2stimulation (S1: six times 150 ms, S2: >5 ms above effective refractory period) to induce VA and waited for at least 3 s to exclude self-terminating VA. Subsequently a 11 s long illumination protocol was performed using four light pulses (**green**, 1 s, 6.5 mW/mm^2) with 1 s in-between and a 4 s long pause after the last light pulse. Termination was analyzed within this 11 s lasting time period in all groups tested. If VA still persisted, we applied VA termination protocols using longer illuminations (6.5 mW/mm^2 for at least 3 s), antitachycardia pacing (50 Hz, > 2s) and/or mechanical defibrillation by hitting the ventricles with tweezers in order to restore sinus rhythm and being able to retest optogenetic defibrillation in this heart. (**C**) Setup for membrane potential recordings in the intact heart (see Fig. 2C-G). Sharp electrodes (**black**) were impaled in the center of the illuminated region (**green circle**, 24 mm^2) ~ 4 mm distant to the pacing electrode until membrane potential recordings were obtained with a resting membrane potential < −60 mV and AP peaks above 0 mV. Electrical pacing was performed at beating rates between 312 and 812 bpm to mimic high frequent VA. 5 s light pulses (7 mW/mm^2) were repeated with 5 s long pauses and AP characteristics were compared before and during illumination.
